# Age-related changes in prepulse inhibition of the startle response

**DOI:** 10.3389/fpsyt.2023.1145783

**Published:** 2023-04-13

**Authors:** Yasmin Guedes de Oliveira, Bruno Costa Poltronieri, Erica Woodruff, Brunno Freitas da Costa, Rogerio Arena Panizzutti

**Affiliations:** ^1^Instituto de Ciências Biomédicas, Universidade Federal do Rio de Janeiro, Rio de Janeiro, Brazil; ^2^Instituto de Psiquiatria, Universidade Federal do Rio de Janeiro, Rio de Janeiro, Brazil; ^3^Instituto Federal de Educação, Ciência e Tecnologia do Rio de Janeiro, Rio de Janeiro, Brazil; ^4^Hospital Universitário Antônio Pedro, Universidade Federal Fluminense, Niterói, Brazil

**Keywords:** prepulse inhibition (PPI), aging, cognition, biomarkers, psychophysiology, healthy older adults

## Abstract

**Introduction:**

Acoustic prepulse inhibition of the startle response (PPI) is a phenomenon characterized by the reduction in the startle reflex caused by the presence of weak and brief stimulus before an intense and sudden stimulus (pulse). These phenomena can be observed in several species, but in humans it is commonly measured by the eyeblink using electromyography. PPI works as an operational measure of sensorimotor gating, which is the ability to suppress motor responses for sensory stimulus. Healthy aging is marked by several changes in neural processing, like inhibitory functioning decline. In this line, PPI measure can be a potential biomarker for changes related to the aging process.

**Methods:**

In this research we aim to investigate if PPI is reduced with aging and if this reduction would be associated with cognitive functioning of older adults. To this aim, we compared PPI levels of older adults (over 60 years old) with PPI levels of young adults (from 18 to 28 years old).

**Results:**

With that, we found, significantly lower PPI level (*F*[1,25] = 7.44 *p* = 0.01) and lower startle amplitude startle amplitude: (U = 26.000 *p* = 0.001) in older adults than in young adults. However, we did not find differences in levels of habituation (T = −1.1 *p* = 0.28) and correlation between PPI and cognition within the sample of healthy older adults.

**Discussion:**

Our results demonstrate that aging is a factor that affects PPI and that it does not seem to predict cognition, however, future studies should explore the potential of using PPI for monitoring cognitive changes associated with techniques such as cognitive training.

## 1. Introduction

In the past decades, efforts have been made in searching ways of measuring the decline of brain functions that can occur in aging. Beyond the classical approach of observing clinical features, the use of biomarkers can be a mechanism of knowing the aging process before it is noticed by a patient or its examiner. This can lead to earlier diagnosis, even in presymptomatic stages of neurodegenerative diseases. The dosage of neurospinal fluid substances and the use of image methods are some of the most commonly used biomarkers for this purpose (1). However, these are invasive procedures that demand a high cost, making their application difficult in the clinical context, as well as in regions with difficult access to this type of tool. Many research efforts have been focused on developing biomarkers that are less expensive, less or non-invasive, and easier to apply. In this context, measuring the prepulse inhibition (PPI), which is the suppression of a startle reflex to an intense stimulus when a weak prepulse stimulus precedes the startle stimulus (2), can emerge as a potential biomarker attached with aging.

Startle reflex is a whole body muscle twitch response that occurs immediately after a sudden intense sensory stimulus (pulse), usually measured in humans as an eyeblink response using electromyography (EMG). This response can be reduced after repeated presentation of the pulse stimulus, causing habituation to the pulse. When a weaker sensory stimulus is presented between 30 and 500 ms before the pulse (prepulse), the startle reflex tends to be reduced in healthy individuals in normal conditions (3), but for conscious identification of the prepulse, the interval between the prepulse and the pulse must be at least 60 ms (4). This phenomenon is called prepulse inhibition (PPI). Braff and Geyer (5) proposed PPI as an operational measure of sensorimotor gating. Sensorimotor gating is the ability of the central nervous system to suppress motor response by filtering irrelevant sensory information (5–7). This normal inhibition ability is commonly impaired in disorders associated with deficits in sensorimotor gating, such as Obsessive Compulsive Disorder (8), Bipolar disorder (9), Huntington disease (10), Panic disorder (11), and symptoms of nocturnal Enuresis in children (12, 13). PPI may not be the best tool to diagnose specific disorders as many conditions associated with impairments in sensorimotor gating can show deficits in PPI. However, it can be an interesting complementary tool for monitoring cognition and changes associated to cognitive status on pathological and non-pathological conditions. It is known that PPI occurs regardless of attentional focus, memory or motivation. However, directed attention was also associated with PPI modulation (14). This attentional mechanism can enhance PPI, but for conscious identification of the prepulse, the interval between the prepulse and the pulse could be between 60 and 140 ms (15–17).

Throughout the aging process, older adults experience a period of development marked by neurobiological and cognitive changes, even in the absence of psychiatric or neurological disorders (18). Often the neurofunctional deficits associated with natural aging are still not clinically detectable and the signs are not visible. In these cases, PPI can be a valid tool to monitor such changes, helping to understand the effects of age on cognition and brain functioning. Few studies were dedicated to investigate the effects of aging on the phenomena associated with startle reflex (2, 3, 19). Different from PPI, that refers to a decrease in response to a stimulus due to the presence of a weaker stimulus previously presented, habituation is defined as a decrease in responding to an initially novel stimulus when it is presented repeatedly at rates slow enough, producing a sensory adaptation (3). This is another inhibitory phenomenon that can be observed and will be used in the present study.

To understand the impact of the aging process in neurophysiological measures is relevant to point to the possibility of using the PPI measure to monitor these changes. The maturation of the ability to modulate the startle response begins after the third year of age, with no significant modulatory ability being observed before that (19, 20). Levels of acoustic startle reflex maturation seem to increase throughout childhood and adolescence (6–18 years) (19). One of the main studies about the age effects on PPI and startle reflex, involved individuals from 18 to 88 years old. It showed that, with normal aging, a decrease in the magnitude startle and an increase in the startle latency was observed. Besides, with regard to PPI, the same authors demonstrated an inverted U-shaped function with age (greatest PPI at intermediate ages) and no differences were found in habituation between age groups (3).

Some studies have also shown differences between young and older adults with regard to the PPI and the startle reflex; however, the results varied according to the paradigm used (21, 22), showing higher inhibition with longer pre-stimulus in both age groups. Nevertheless, in young adults, from 80 ms on, there was a reduction in inhibition, while in old adults, inhibition continued to increase until the pre-stimulus of 120 ms (21). With regard to the relationship between PPI and cognitive functioning, PPI was positively correlated to performance in different cognitive functions, such as attention, working memory, planning ability, and strategy formation (23–28). Studies with patients diagnosed with schizophrenia sought to correlate the PPI with the symptoms and functionality of the patients, in addition to investigating the use of this parameter as a measure of treatment response (29, 30). Such results suggest that although the PPI evaluation is not the best tool to diagnose specific disorders, it can be an interesting biomarker to evaluate the effect of treatments (31). Among some of the advantages of using the PPI as an evaluation tool are the fact that it does not depend on the motivation of the evaluated subject, as it is a pre-attentive measure, in addition to being a sensitive measure to changes caused by drugs, sensory and cognitive manipulations (32). Regarding the study of healthy older adults, there are no published studies that demonstrate a correlation between PPI and cognitive functioning or that investigate changes in PPI associated with cognitive or pharmacological interventions.

Older adults experience a period of development characterized by neurobiological and cognitive changes. Even in the absence of psychiatric disorders or neurological diseases, often the neurofunctional deficits associated with physiological aging are not clinically detectable and the signs are not visible. Considering these aspects, the PPI could be a possible tool to monitor such changes, helping to understand the effects of age on cognition and brain functioning. Thus, this work aims to investigate the impact of aging on prepulse startle inhibition response and on pulse habituation, in order to understand how this phenomenon could be associated with cognitive changes common to aging. Our hypothesis is that healthy older adults have lower PPI compared to young people and that PPI levels may be associated with cognitive performance in older adults.

## 2. Materials and methods

### 2.1. Participants

The present comprised two different age groups. The first one was composed of 14 healthy older adults (mean age of 71; SD 6.45) of both sex (12 women and two men), recruited from Senior Centers of the city hall of Rio de Janeiro. Inclusion criteria were: Participants were aged 60 years or older, fluent in Portuguese, literate, without neurological and neuropsychiatric disorders, and had a score greater than or equal to 23 in the Mini Mental State Examination, a score equal to or greater than 70 in the Intelligence quotient (Matrix Reasoning and Information subtests of the Wechsler Scale for Adults), and up to four points on the Geriatric Depression Scale (GDS). All older adults underwent an initial audiometry, cognition, and functionality assessment. These participants were excluded if they had hearing threshold greater than 40 db, neurological or psychiatric disorders.

The second one was composed of 14 young adults between the ages of 18 and 28 of both sex (11 women and three men) from two universities located in the city of Rio de Janeiro, mostly undergraduate students. They were excluded if they had neurological or psychiatric disorders, in addition to a hearing threshold greater than 40 db.

More information about Sociodemographic and clinical characteristics of the sample can be checked in Table 1. Some information like smoking, menopause, medications, and recently surgery on the face were collected. None of the older adults participants smoked and one young adult was a smoker, but he informed his last cigarette was around 3 h before the assessment. The study was approved by the Research Ethics Committee of the Clementino Fraga Filho University Hospital of the Federal University of Rio de Janeiro (HUCFF-UFRJ) under number 17976113.10000.5257.

**Table 1:** Sociodemographic data about older adults and young adults.

Sociodemographic and clinical characteristics of participants			
	Older adults (*N* = 14)	Young adults (*N* = 14)	*p* value
	Mean (Standard deviation)	Mean (Standard deviation)	
Age (years)	71 (6.45)	23.23 (2.43)	<0.001*
Gender (Female/Male)	12/2	11/3	0.62
Education (years)	17.29 (2.89)	12.42 (1.44)	0.04*
Marital status (Single/Married/Divorced/Widowed)	0/4/6/4	14/0/0/0	<0.001*
Smoke status (Smoker/Non-smoker)	(0/14)	(1/13)	0.3
Menopause	12	-	
MMSE	28.4 (1.65)	-	
I.Q	114.4 (12.69)	-	
GDS-15	2.64 (2.2)	-	

MMSE, mini mental state examination; GDS, geriatric depression scale. T-test revealed differences in age (T = −25.9; *p* ≤ 0.001) and education (T = −5.01, *p* = 0.04) between groups. Chi-square revealed no difference in gender between groups: *X*^2^ (1, *N* = 28) = 0.24, *p* = 0.62 and Smoke status: *X*^2^ (1, *N* = 28) = 1.04, but significant difference in marital status between groups: X^2^ (3, *N* = 28) = 24.8, *p* = <0.001.

### 2.2. Audiometry

The older and younger adult groups were assessed using the “Audio Check” program,^1^ available for free online. The assessment was performed in a silent room with background noise of 30–40 db. To determine the hearing threshold of each participant, we used the frequencies of 125, 250, 500, 1,000, 2,000, 4,000, and 8,000 Hz. To detect the degrees of hearing loss, we performed the calculation of the hearing threshold proposed by the WHO (33), which considers the average of the hearing threshold in the frequencies of 500, 1,000, 2,000, and 4,000 Hz. Thus, normal hearing is considered to be an average of 0–25 dB, mild loss of 26–40 dB, moderate loss of 41–60 dB, severe loss of 61–80 dB, and profound loss above 80 dB (34). For this study, we determined the auditory threshold of up to 40 dB, configuring mild loss (ability to hear and repeat words at normal volume at a distance of 1 m), due to the fact that we did not have the possibility of performing the audiometry in an environment with total acoustic isolation, relying on the isolation provided by the headphone, same as used on PPI assessment.

Due to the common hearing impairment associated with aging, we performed an audiometry to exclude individuals who had moderate hearing loss, because this aspect could influence the PPI. Furthermore, we compared the average auditory threshold of older adults (mean = 17.95) with young adults (mean = 11.4) and we observed a greater hearing threshold in older adults compared to young people, U = 34.5, *p* = 0.002.

### 2.3. Eyeblink response monitoring

Electromyography of the orbicularis oculi was monitored, digitized, and analyzed with the Biopac MP150 (Biopac Systems Inc., Goleta, CA, United States). The signal was integrated using AcqKnowledge 5.0 software, and the maximum response to each pulse (120 dB) was accounted for using 50 ms epochs, during a 100 ms window. Subjects were assessed in a sound-attenuated room. Two EL254S electrodes (Biopac Systems) filled with conductive gel were connected to the subjects’ left orbicularis muscle to capture the startle response of eye blinks, through the EMG 100C module (Biopac Systems). The left side was chosen following the recommendation of a guideline about human startle eyeblink electromyographic studies (35). One ground electrode was placed on the forehead. Electrical signals were amplified (2,000x) and filtered using a passband of 10–500 Hz.

### 2.4. Cognitive assessment of older adults

Eligible participants performed an assessment of the following cognitive functions with their respective tests: Attention (Identification task of the CogState computerized battery and Trail Making Test—A); Processing speed (Detection task of the CogState computerized battery); language (verbal fluency test—animals category); memory and learning (RAVLT—Rey Auditory Verbal Learning Test and Cambridge Cognitive Examination—CAMCOG Recall); cognitive flexibility (CogState computerized battery Set-Shifting tasks and the Trail Making Test—difference between part A and part B); and social cognition (Social Emotional Cognition task of the CogState computerized battery).

### 2.5. PPI assessment of older adults and young adults

In the first experiment we used white noise prepulses (mixture of all audible frequencies, comprising from 20 to 20,000 Hz) at 75 dB/60 ms, 75 dB/120 ms, 85 dB/60 ms, and 85 dB/120 ms before the white noise pulse with intensity of 120 dB. Interstimulus intervals ranged from 11 to 25 s. Startle response was measured within 100 ms of pulse presentation. Considering the known difficulty of older people in processing higher frequency sounds, we performed a second experiment on a part of our sample, except three participants from the first experiment (see Supplementary Data), in order to evaluate the possible differences in inhibition percentage in older and younger adults, using prepulses of low and high frequencies, 500 Hz and 4 kHz.

The second experiment was implemented right after three participants had been evaluated in the first one. Thirteen older adults (see Supplementary Table) with mean age of 70.3 years (SD = 6.03) and 12 young with mean age of 22.9 years (SD = 2.2). Both experiments were performed on the same day, with an interval of 5 min between them. As a passive paradigm, participants were instructed just to keep their eyes closed while a sound was presented and no action was needed.

In the first experiment, 10 samples of each type of event were randomly presented for 22 min: white noise pulses preceded or not by one of four different intervals and intensities of prepulses (Pulse alone, Prepulse of 75 dB/60 ms + Pulse, Prepulse of 75 dB/120 ms + Pulse, Prepulse of 85 dB/60 ms + Pulse, and Prepulse of 85 dB/120 ms + Pulse) with the first 5 min being the noise habituation period 65 dB white noise. After that time, five pulses (initial pulse) alone were presented before the random presentation of the pulses with and without prepulses. The last five pulses (final pulse) were also presented alone to calculate habituation, which was expressed as the percentage of inhibition of the mean startle amplitude in response to the presentations of the final five pulses, as a function of the mean amplitude of the response to the initial five pulses. The second experiment lasted 14 min and did not contain the five initial pulses and the five final pulses to assess habituation. Prepulses of 500 Hz and 4 kHz were presented randomly preceding white noise pulses. In order to reduce interference from external noise, the experiment took place in a quiet room and the audio was presented through a “Gamer Razer Kraken Headset” with high acoustic isolation capacity. The capture of electrical signals of the startle response was carried out from a passive paradigm, that is, the subjects were instructed to not perform any task during the experiment, in addition to keeping their eyes closed until the end of the presented audio. Stimuli were the same for all participants and presented in the same order.

### 2.6. Statistical analysis

Statistical data analyses were performed using the IBM SPSS-20 software. For characterizing the cognition data of the older adults group, we analyzed the means and SDs of the raw scores and *Z* score of the tests were presented.

Demographic data, such as gender, smoke, and marital status, were analyzed by comparing the two groups using Chi-square (Fisher) analysis, while an independent sample *t*-test was conducted to compare the differences in education years between groups. For clinical characteristics in the older adults group, such as GDS, MMSE, and I.Q., we calculated the means and standard deviations of the participants.

For the auditory threshold, we compared the means between the two groups by using the non-parametric Mann–Whitney test and an independent sample *t*-test was used to compare the audiometry of each frequency between the groups.

To analyze the habituation, we did also perform an independent samples *t*-test in order to compare means of habituation to the pulse between the two age groups. For the magnitude of Startle a Mann–Whitney test was performed to compare means of startle between the two groups. Startle data were treated with 80% winsorization. The mean of startle magnitude of pulse alone was used as covariate in one of ANOVAs analysis to compare PPI between groups. Habituation was expressed by the following formula: % habituation = 100 − [(mean initial pulse/mean final pulse) × 100].

To compare PPI between groups in 65 dB/60 ms, 75 dB/120 ms, 85 dB/60 ms, and 85 dB/120 ms, we used a mixed repeated-measures ANOVA with intensity/intervals of prepulse as within-subject factor and age group as the between-subject factor. While, for the comparison between groups of the PPI with different high (4 kHz) and low (500 Hz) frequencies, an independent samples *t*-test. For correlations between PPI-Age and PPI-Cognition, a Pearson’s correlation test was carried out. For cognition analysis, we used a composite of each cognitive domain’s *z*-score to correlate cognition and PPI. PPI was demonstrated from the percentage reduction in the startle response when a prepulse (PP) was presented 60 or 120 ms before the pulse (P), expressed through the formula {100 − [100 × (PP/P)]}.

Associations between auditory threshold and PPI, startle and PPI, and startle and auditory threshold, were analyzed by Spearman’s correlation. The sample calculation revealed the need for at least 17 participants per group, considering a minimum difference of 20% in PPI between the means of the two populations, standard deviation of 20 and alpha error of 5% and beta error of 20% (80% power).

## 3. Results

### 3.1. Participants’ PPI and correlation between age and PPI

**Figure 1:**
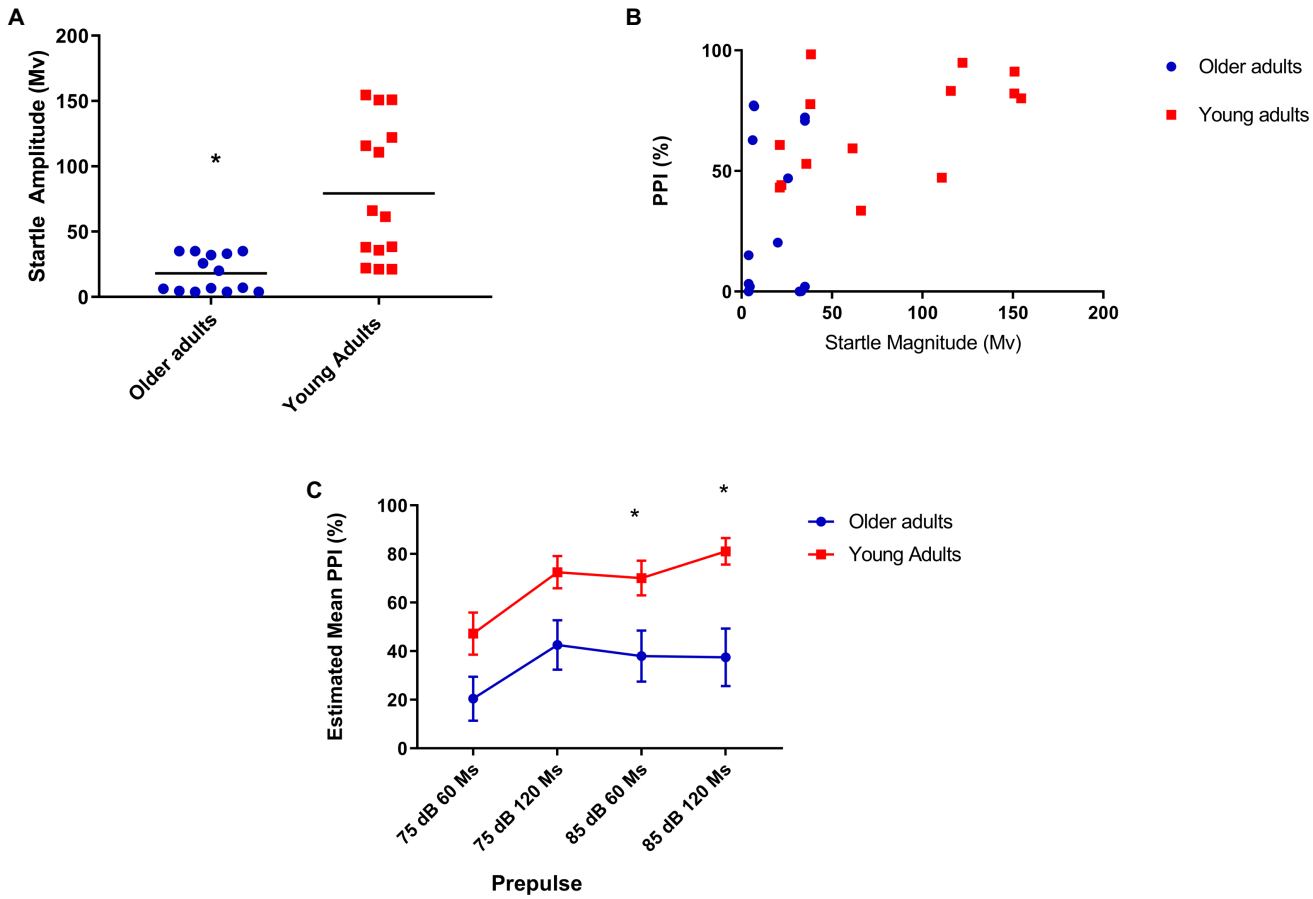
**(A)** Older adults show less startle when compared to young people. Mean startle amplitude: 21.64 (older), 79 (young) U = 26.000 *p* = 0.001; **(B)** Moderate positive correlation between prepulse inhibition and startle in the youth group. Older (*N* = 14), *r* = 0.12, *p* = 0.68; and young (*N* = 14), *r* = 0.53, *p* = 0.05; **(C)** Older adults show less inhibition with prepulse of 85 dB at 60 and 120 ms covariating PPI for startle reflex. Older (*N* = 14) and younger (*N* = 14) Repeated-measures ANCOVA corrected for startle: 85 dB/60 ms = Mean = 26.1 (older), 70.06 (young *N* = 14)—*F*[1,25] = 7.93, *p* = 0.009; 85 dB/120 ms = Mean = 38.4 (older), 81.4 (young)—*F*[1,25] = 7.76 *p* = 0.01.

**Figure 2:**
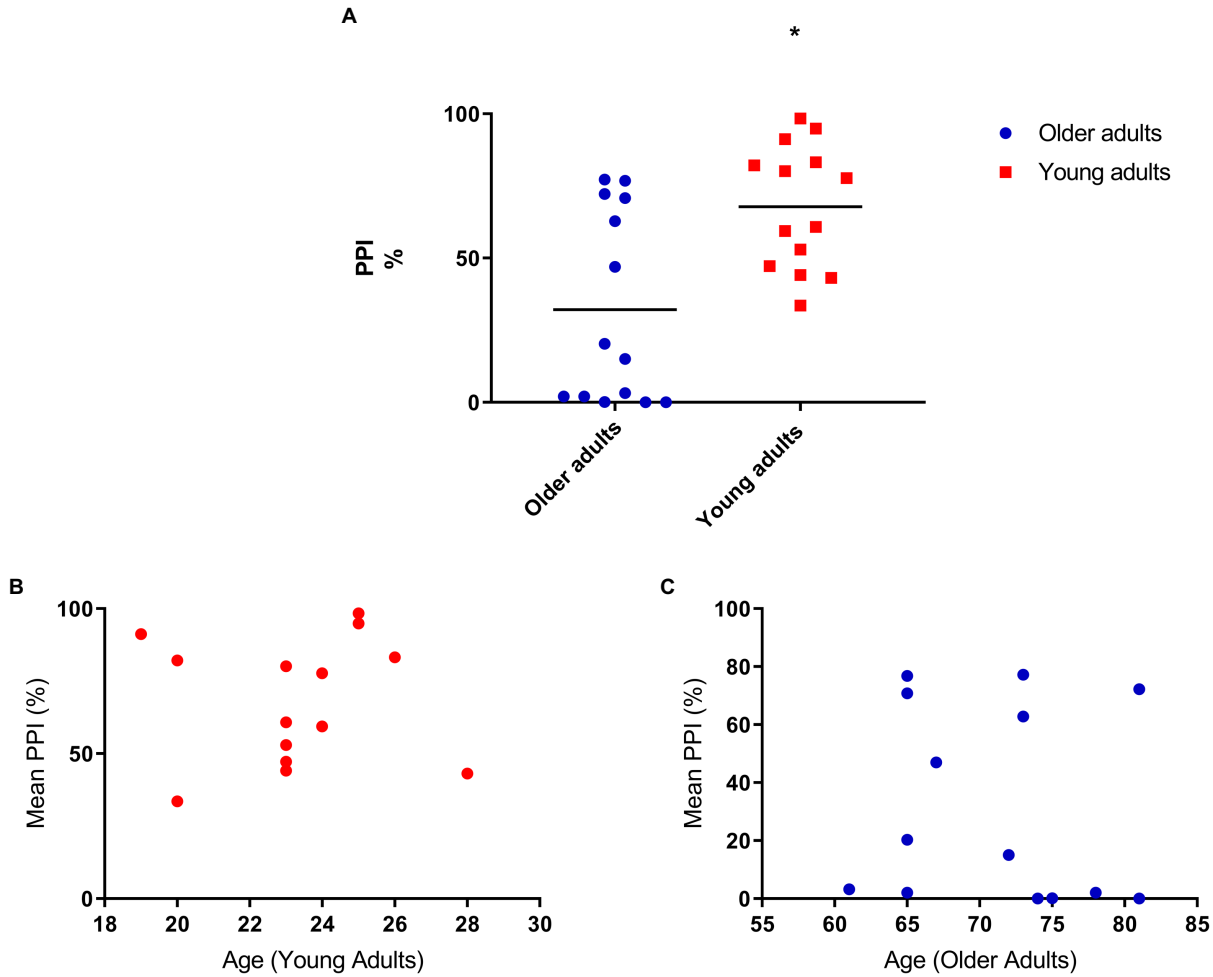
**(A)** Older adults have less prepulse inhibition compared to young adults. Mean percent inhibition: 32.5 (older adults), 67.76 (young adults). ANOVA corrected for startle *F*[1,25] = 7.44 *p* = 0.01; **(B)** Prepulse inhibition does not correlate with age (young adults; *N* = 14), *r* = 0.01, *p* = 0.1; **(C)** Prepulse inhibition does not correlate with age (older adults; *N* = 14), *r* = −0.08, *p* = 0.8.

We performed the PPI assessment and we found a significant difference between the two groups. Because of the difference in the startle response between the two groups (Figure 1A), we analyzed the prepulse inhibition correcting for startle. We still found significant differences in the PPI between the groups: *F*[1,25] = 7.44 *p* = 0.01 (Figure 2A). For this reason, we did a correlation to evaluate if these differences would be associated with age and when analyzed separately the groups, we found no significant correlation: *r* = −0.08 *p* = 0.8 (Figures 2B,C).

### 3.2. Hearing threshold, audiometry, correlation PPI × hearing threshold, and PPI between de groups

**Figure 3:**
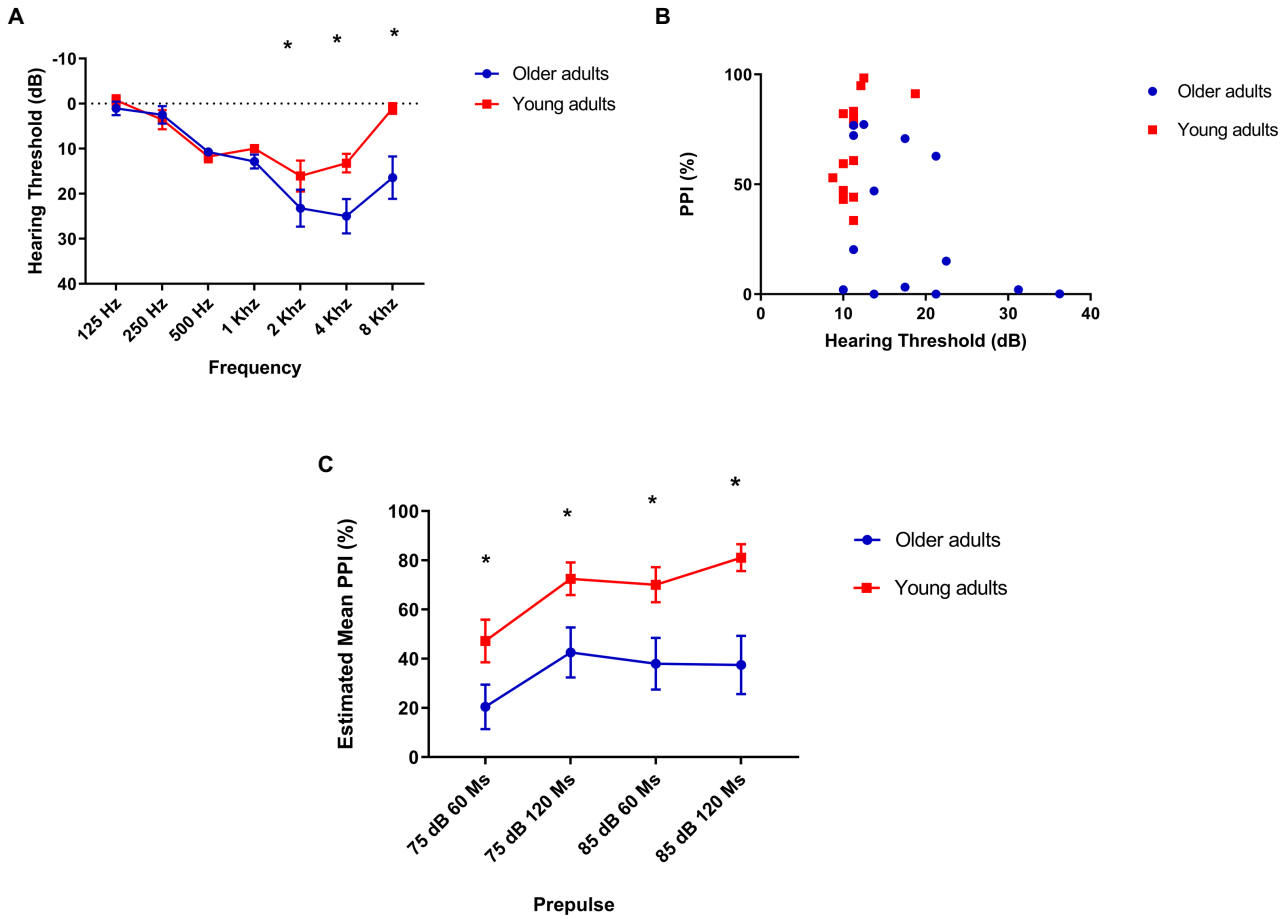
**(A)** Older adults have a higher hearing threshold at 2, 4, and 8 kHz. The lines indicate average loss in decibels at each frequency. 2 kHz: mean 13.21 (young), 23.21 (older) *t* = −2.38, *p* = 0.025. 4 kHz: mean 12.14 (young), hearing threshold of older adults compared to older adults young. Mean = 17.95 (older), 25 (older) *t* = −3.1, *p* = 0.005. 8 kHz: mean 1.07 (young), 16.4 (older) *t* = −3.17, *p* = 0.04; **(B)** There is a moderate, but not statistically significant correlation between prepulse inhibition and hearing threshold in older adults: Older adults (*N* = 14) Spearman’s Rhô (ρ) = −0.45, *p* = 0.1 and young (*N* = 14) Spearman’s Rhô (ρ) = −0.61 *p* = 0.02. **(C)** Older adults show less prepulse inhibition than young people at all intervals and intensities tested. Repeated-measures ANOVA found no within-subject interaction (interval/intensity of prepulse + age) effect on PPI: *F*[2.3, 61.12] = 8.7 *p* = 0.33, but a simple effect of interval/intensity of prepulse was found: *F*[2.3, 61.12] = 8.7 *p* < 0.001. *Post hoc* analysis showed that young adults group had significantly lower PPI in 75 dB/60 ms than 75 dB/120 ms (*p* = 0.017), 85 dB/60 ms (*p* = 0.04), and 85 dB/ 120 ms (*p* = 0.005) as older adults had significantly lower PPI in 75 dB/60 ms than in 75 dB/120 ms. Older (*N* = 14) and younger (*N* = 14). Repeated-measures ANOVA revealed a significant effect of age between groups on PPI in every interval/intensity of prepulse tested: *F*[1,26] = 11.3 *p* = 0.002. 75 dB/60 ms: Mean = 21.36 (older), 47.23 (young *N* = 14)—*F*[1,26] = 4.56, *p* = 0.042; 75 dB/120 ms: Mean = 44.06 (older), 72.48 (young)—*F*[1,26] = 6.07 *p* = 0.021; 85 dB/60 ms: Mean = 26.1 (older), 70.06 (young)—*F*[1,26] = 11.02 *p* = 0.003; and 85 dB/60 ms: Mean = 38.4 (older), 81.4 (young)—*F*[1,26] = 11.25 *p* = 0.002.

To detect hearing loss, we were interested in evaluating the difference between both groups in different frequencies, since we assessed the inhibition with prepulses in low (500 Hz) and high (4 kHz) frequency in comparison with young adults (Figure 3A). Our assessment showed that older adults have a higher hearing threshold at 2 kHz [mean: 13.21 (young), 23.21 (older) *t* = −2.38, *p* = 0.025], 4 kHz [mean: 12.14 (young), 25 (older) *t* = −3.1, *p* = 0.005], and 8 kHz [mean: 1.07 (young), 16.4 (older) *t* = −3.17, *p* = 0.04].

As this is a comparison of two different age groups, in which the oldest is associated with hearing loss, we were interested in confirming whether the lowest performance on the PPI would be associated with hearing. However, we found a moderate negative correlation, but not statistically significant between auditory threshold and PPI in the group of older adults: Spearman’s Rhô (ρ) = −0.45, *p* = 0.1 (Figure 3B).

We found a significant difference in PPI in all intensities of prepulse intervals between groups, with older adults showing less PPI than young adults: *F*[2.34, 58.6] = 11.3 *p* = 0.002 (Figure 3C).

### 3.3. Habituation, startle magnitude, and PPI

The habituation to the sound pulse could influence the PPI, since a quick habituation in one of the groups could be associated with possible differences in the percentage of inhibition between the two populations. With higher percentages indicate more habituation, in this case, there is no difference in habituation related to the pulse between both groups. Mean habituation percentage in young adults was 74.92% and in older adults was 82.9%. Independent samples T-test revealed no significant difference between groups: T = −1.1 *p* = 0.28.

Afterward, we compared the startle amplitude means of the two groups and identified a difference between the startle means of the older adults and young adults groups (Figure 1A). Due to the significant difference between the startle response of the two groups, we did a correlation test to assess if this difference could be associated with the mean auditory threshold, but we found no correlation between the two measurements in both age groups Spearman’s Rho (ρ) = −0.031, *p* = 0.92. Based on differences found in startle magnitude, we performed an analysis of PPI using subgroups matched for startle magnitude, generating two subgroups: One containing the lowest startling 50% of younger subjects and another containing the highest startling 50% of older subjects. Interestingly, the smaller sample size prevented detecting significant PPI differences similar to total sample: Mixed ANOVA revealed a significant effect of age group on PPI between groups: *F*[1, 2] = 12.8, *p* = 0.004, *N* = 14 (Smaller sample size) vs. *F*[1,26] = 11.3, *p* = 0.002, *N* = 28 (Total sample), See Supplementary Figure 1.

As this is a comparison of two different age groups, in which the oldest is associated with hearing loss, we were interested in confirming whether the lowest performance on the PPI would be associated with hearing. However, we found no correlation between auditory threshold and PPI in the group of older adults. Due to the differences found between the two groups in the startle response and in the PPI, we performed a spearman test which found a positive correlation between the two measures in the group of young people. There is a positive correlation for the young adult’s group as it is possible to see in Figure 1B.

Initially, we found a significant difference in PPI at all intensities prepulse intervals between groups, but when analyzing PPI correcting for startle, we found a significant difference in PPI between groups in the presence of prepulses of 85 dB/60 ms before the pulse: *F*[1,25] = 7.93, *p* = 0.009, and 120 ms before the pulse: *F*[1,25] = 7.76, *p* = 0.01 (Figure 1C).

### 3.4. PPI with prepulse of 500 Hz and 4 kHz

**Figure 4:**
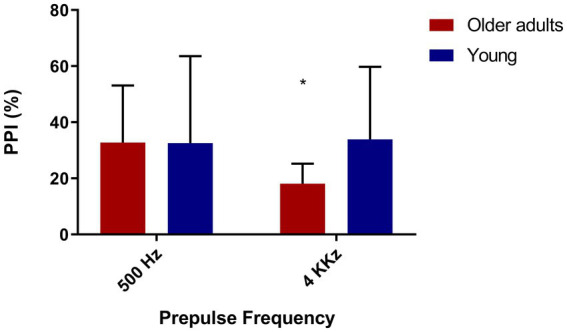
Older adults show less inhibition with high frequency prepulse. Older adults (*N* = 12), young people (*N* = 13). 500 Hz: *t* = 1.41/*p* = 0.17—Mean = 68.25(younger), 53.55 (older). 4,000 Hz: *t* = 4.44/*p* = 0.00—Mean = 62.46 (young), 14.98 (older).

We evaluated PPI in a part of the sample (*N* = 25) with the presence of prepulses in different frequencies (low and high), in order to verify if there were differences in PPI between the two groups according to the kind of frequency in the prepulse. As expected, we observed lower PPI in old adults in the presence of a high frequency prepulse (4 kHz; Figure 4).

Since we found significant differences between the groups in PPI with a prepulse of 4 kHz, we sought to assess if this could be associated with hearing; however, we found no correlation between the 4 kHz PPI and the auditory threshold (loss in decibels), Spearman’s rhô (ρ) = −0.09, *p* = 0.68.

Lastly, due to the difference in the audiometry of 4 kHz between both groups and in PPI with prepulse of 4 kHz, we investigated if these two measures correlate with each other, but we did not find any correlation, Spearman’s rhô (ρ) = −0.005, *p* = 0.98.

### 3.5. Correlation between PPI and cognitive performance

To assess whether prepulse inhibition was associated with the cognitive functioning of older adults, we use a correlation between cognitive domains tested and PPI; however, there is no significant correlation between PPI and cognition (Table 2).

**Table 2:** There is no correlation between PPI and cognitive performance by older adults.

Correlation between PPI and performance in the cognitive tests		
Domain	*R*	*p* value
Attention	−0.062	0.83
Verbal fluency	0.19	0.51
Processing speed	0.17	0.56
Praxis	0.46	0.1
Social cognition	−0.34	0.23
Cognitive flexibility	−0.08	0.8
Recall	−0.3	0.3
Retention	−0.12	0.68
Learning	0.39	0.17
Memory	−0.38	0.16

Pearson’s correlation did not reveal any significant correlation between cognition and PPI. *N* = 14 older adults.

## 4. Discussion

The present study aimed to understand the effects of aging on PPI. For this purpose, we evaluated the difference in the PPI of two different age groups: Young (mean age of 23) and old adults (mean age of 71). We found a higher level of PPI in the young adults group, demonstrating that aging may be associated with lower levels of PPI. These findings are distinct from some previous studies, which did not demonstrate differences in mean PPI of older and younger adults, (4, 36), although a previous study demonstrated significantly more PPI in ages between 36 and 50 years old, while the age group comprising people between 18 and 24 years old showed no significant differences compared to the group of older adults aged 58 and 88 years old (3). But in line with some mouse studies that observed lower levels of PPI in older mice compared with young ones (37). However, in some other previous clinical studies, the PPI level of older adults differ according to emotional and attentional conditions (4, 38, 39). In this study, though, we focused only on the pre-attentive aspects of the PPI assessment, using a passive assessment paradigm, measuring eyeblinks to sound stimuli, which did not involve any additional cognitive task or emotional condition.

In our sample, older adults showed significantly reduced startle compared to young adults, in line with previous studies that demonstrated reduced startle both in humans and in rats and mice when comparing older subjects with young ones (36, 40, 41). As we found a correlation between startle and PPI, we performed a covariate analysis for startle, which maintained the significance of percentage inhibition differences between groups in 85 dB/60 ms and 85 dB/120 ms. The two evaluated groups did not show differences in the level of habituation, as in previous studies (3).

Our results showed that hearing threshold did not affect PPI and therefore we consider that the lower levels of PPI found in older adults in this study may be not directly associated with hearing loss commonly found in older people. Another important point to highlight is that, although there is a difference between the hearing threshold of the group of older adults and the group of young ones, the means are classified in the same way according to the WHO standards for audiometry: hearing threshold lower than 25 dB does not indicate hearing loss (33).

Older adults had a higher hearing threshold at the 4 kHz frequency than younger adults, which made us imagine that the lower levels of PPI with prepulse at 4 kHz compared to PPI with prepulse at 500 Hz could be associated with this loss. Despite this, we found no correlation between PPI and auditory threshold at this same frequency. This may lead us to think that, in our sample, even though prepulse detection at high frequencies has occurred, there may have been difficulties in processing the pulse. As raised in the initial hypothesis, we found a significant difference in PPI between the groups with a prepulse frequency of 4 kHz, curiously, there was no significant difference between the groups in the frequency of 500 Hz.

The phenomenon of prepulse inhibition is mediated by several neural pathways, from structures of the auditory system, the limbic system, and superior cortical areas (41). A recent systematic review has shown that thalamic, striatal, and frontal lobe are the most evidenced (9 studies) areas activated in healthy populations during PPI (17). Aging, in general, is associated with physiological changes that can affect the normal functioning of some of these structures, and this may be related to many of the results we found in our study. In the case of reduced startle response in older adults, we know that even individuals who have peripheral auditory integrity may present impairments in central auditory processing, from the cochlear nucleus (42). Furthermore, the lower PPI found in older adults may be related to the reduction of common auditory input in aging (43). However, the similar results found when comparing the habituation percentage of older and younger adults suggests that this kind of short-term neuroplasticity remains preserved with aging.

One of the main limitations of the study is the low number of evaluated subjects (14 participants in each group), in addition, more sensitive tests were not used to assess the ability to inhibit responses, which could be more associated with the ability to inhibit distractors.

Only older adults performed a cognitive assessment, while the younger group only answered the mini questionnaire from DSM-V, to exclude participants with mental disorders. Therefore, we could not assess whether the younger ones actually had a better cognitive profile than the older adults who participated in the study.

Another point to be considered as limitation is the possible influence of hormonal factors like menstrual cycle and hormonal contraception, although most of the participants in the present study were women the information about the menstrual cycle was not collected. Previous studies have shown that menstrual cycle, for example, is a factor that impacts PPI levels (44). Another recent study investigating sex differences on PPI and contraceptive use revealed higher PPI in men than in women who do not use contraceptives; however, no significant differences were found between women who use and women who do not use contraceptives (17). According to previous studies, lower PPI of older adults would not be explained by this factor, since the older women had already gone through the menopause period and only the pre-menopause period had been associated with lower PPI level (45). In our study, sex differences could have more influence if the groups were not balanced regarding the gender of the participants. Another potential limitation of this study is that although we have collected the information about drug use, caffeine, and nicotine, the participants were not instructed to avoid caffeine before the assessments.

To understand the impact of PPI on the aging process, it would be interesting to include participants from other age groups, observing whether there are changes in PPI comparing a larger number of subjects from different age groups, from the youngest to the oldest.

Findings from the PPI assessments of the older and younger adult groups lead us to conclude that the aging process is associated with a reduction in PPI levels. The lack of correlation between hearing and PPI indicates that the deficits found in the PPI of older adults compared to young ones are not necessarily related to hearing ability. Although the significant differences between the two groups in audiometry do not indicate any degree of hearing loss for the older adult group.

The current study findings seem promising, as they show that there are differences in PPI associated with healthy aging, indicating that there is room for observing changes in this neurophysiological response. Future studies that assess the impact of pharmacological or behavioral interventions on prepulse inhibition in healthy older adults are needed, as well as studies that involve the relationship between cognitive functioning and PPI using neurocognitive testing that more specifically assess inhibitory functions and auditory processing. In view of the results of the present study, PPI seems to be an interesting biomarker for changes associated with aging.

Our main interest was to investigate whether, in fact, there was any reduction in the PPI of cognitively healthy older adults and, therefore, whether there was room for an increase in PPI. We found differences in PPI between the two investigated age groups, which were not necessarily due to hearing impairment. Therefore, taking age into account when using PPI as a biomarker in studies in different contexts is important, since age is a factor that has been shown to significantly affect the level of inhibition, regardless of differences in startle and auditory threshold.

## Data availability statement

The original contributions presented in the study are included in the article/Supplementary material, further inquiries can be directed to the corresponding author.

## Ethics statement

The studies involving human participants were reviewed and approved by the Research Ethics Committee of the Clementino Fraga Filho University Hospital of the Federal University of Rio de Janeiro (HUCFF-UFRJ). The patients/participants provided their written informed consent to participate in this study.

## Author contributions

YO and RP were involved in the conception and conduction of the study. YO conducted statistical analyses. RP oversaw project development and provided data interpretation. YO, BP, EW, and BC wrote the manuscript. All authors contributed to the article and approved the submitted version.

## Funding

This work was supported by the National Institutes of Health—Fogarty International Center (Grant R03TW009002 to RP); Fundação de Amparo à Pesquisa do Estado do Rio de Janeiro (FAPERJ) (Grant E-26/110.305/2014 to RP); and Conselho Nacional de Desenvolvimento Científico e Tecnológico (CNPq; Grant 400455/2012–9 to RP). RP is an Atlantic Fellow of the Global Brain Health Institute.

## Conflict of interest

The authors declare that the research was conducted in the absence of any commercial or financial relationships that could be construed as a potential conflict of interest.

## Publisher’s note

All claims expressed in this article are solely those of the authors and do not necessarily represent those of their affiliated organizations, or those of the publisher, the editors and the reviewers. Any product that may be evaluated in this article, or claim that may be made by its manufacturer, is not guaranteed or endorsed by the publisher.

## Footnotes

^1^www.audiocheck.net
