# Supplementary material for: Age-related changes in prepulse inhibition of the startle response
Source: Front Psychiatry. 2023 Apr 13;14:1145783. doi: 10.3389/fpsyt.2023.1145783 (PMC10133574; doi:10.3389/fpsyt.2023.1145783)
Supplement: Supplementary file 1 [file Data_Sheet_1.PDF]

Supplementary Figure 1: Comparing 50% of older adults sample with higher startle and 50% of young adults with lower startle

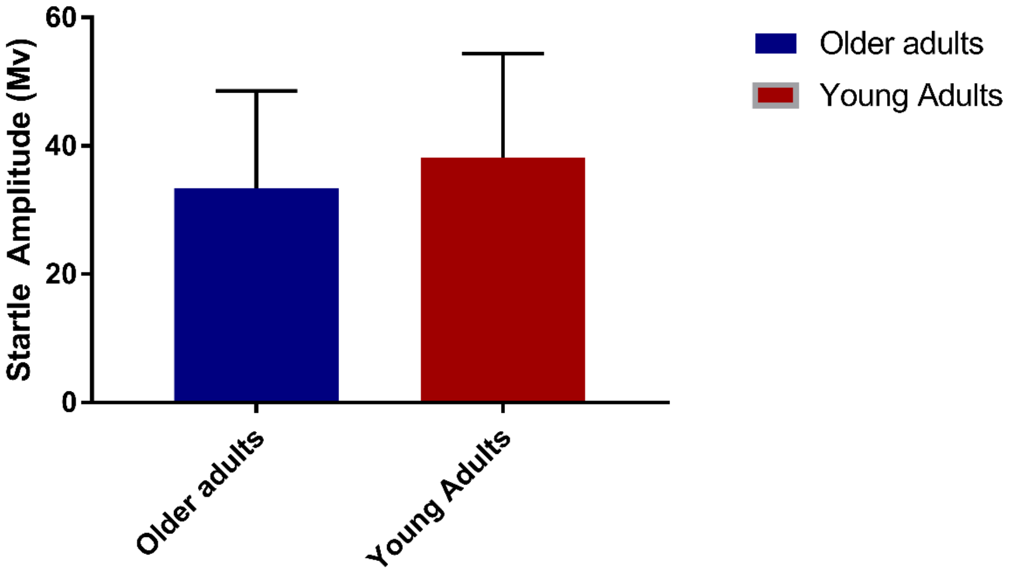

Mann Whitney did not reveal differences in startle between groups N= 14. Mean= 33.4(SD=24.1)Young adults (N=7), Mean 38.17 (SD= 31.4)Older adults (N=7). U= 23 P= 0.85

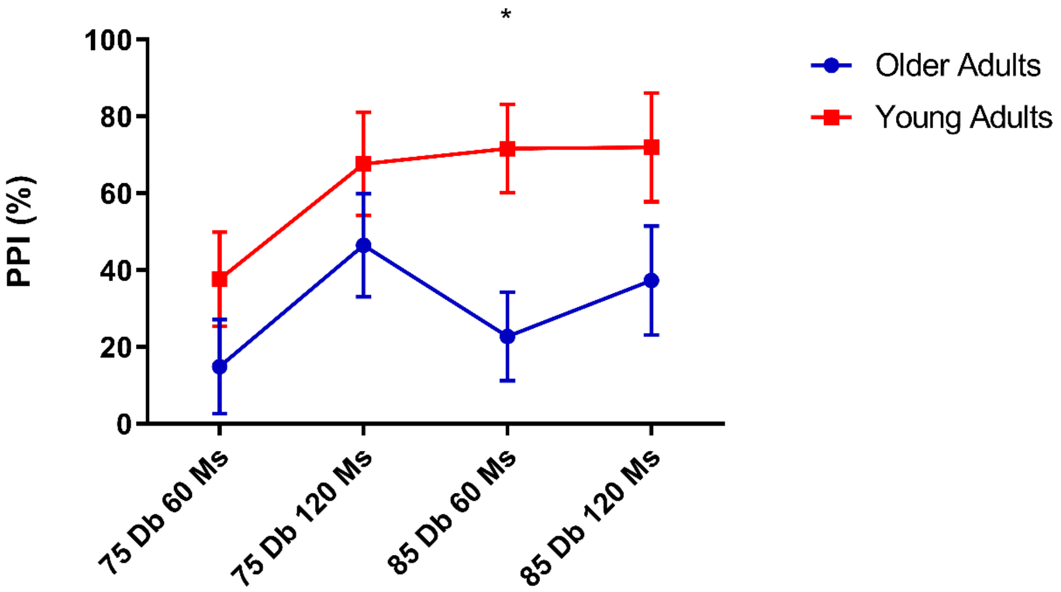

ANOVA found no within - subject interaction (interval/intensity of prepulse + age) effect on PPI: F[3,36]= p=0.08. Mixed Anova revealed a significant effect of age group on PPI between groups: F[1, 12]=4.92.8 P= 0.04. Repeated measure Anova: 75db 60ms: Mean = 14.9 (older), 37.66 (young N=7) – F[1.12] = 1.73, p= 0.2; 75 db 120 ms: Mean= 46.47 (older), 67.64 (young)

– $F[1,12] = 1.24$ $p= 0.017$; 85 db 60 ms: Mean= 22.74 (older), 71.64 (young) – $F[1.12]= 8.98$ $p= 0.01$; 85 db 120 ms: Mean=37.28 (older), 71.96 (young) – $F[1,12]= 3$ $p= 0.1$.
