# Supplementary material for: Age-related changes in prepulse inhibition of the startle response
Source: Front Psychiatry. 2023 Apr 13;14:1145783. doi: 10.3389/fpsyt.2023.1145783 (PMC10133574; doi:10.3389/fpsyt.2023.1145783)
Supplement: Supplementary file 2 [file Table_1.docx]

| **Sociodemographic and clinical characteristics of participants of frequency PPI** | | |  |
| --- | --- | --- | --- |
|  | **Older adults (N=13)** | **Young adults (N=12)** | **P - value** |
|  | **Mean (SD)** | **Mean (SD)** |  |
| **Age (years)** | 70.3 (6.03) | 22.9 (2.2) | <0.001 |
| **Gender (Female/ Male)** | 12/1 | 9/3 | 0.24 |
| **Education (years)** | 17.5 (2.9) | 12.6 (1.5) | 0.04 |
| **Marital Status**  **(Single/Married/Divorced/Widowed)** | 0/4/5/4 | 12/0/0/0 | <0.001 |
| **MMSE** | 28.8 (1.4) | - |  |
| **I.Q** | 116.1 (12.6) | - |  |
| **GDS-15** | 2.7 (2.5) | - |  |

In this table only the data of the participants who participated in the second experiment are presented. T-test revealed differences in age (T=-25,7; P=<0.001) and education (T=-5.17, P= 0.4) between groups. Chi Square revealed no difference in gender between groups: X² (1, N=25) = 1.4 P=0.24, but significant difference in marital status between groups: X² (3, N=25) = 25 P=<0.001. Participant’s characteristics and sociodemographic data are very similar to the overall sample.
